# A multi-center, randomized, double-blinded, parallel, placebo-controlled study to assess the efficacy and safety of Shenqisuxin granule in complex coronary artery disease after PCI: Study protocol

**DOI:** 10.3389/fcvm.2022.1000379

**Published:** 2022-09-12

**Authors:** Xiaoping Wu, Mingyu Yan, Xingxue Pang, Hui Wu, Zhigeng Hu, Rui Xiao, Jianlue Pan, Ying Li, Shengnan Shi, Yanping Deng, Jiaxi Li, Peili Wang, Keji Chen

**Affiliations:** ^1^National Clinical Research Center for Chinese Medicine Cardiology, Xiyuan Hospital, China Academy of Chinese Medical Sciences, Beijing, China; ^2^Graduate School of China Academy of Chinese Medical Sciences, Beijing, China; ^3^Graduate School of Beijing University of Chinese Medicine, Beijing, China; ^4^Cardiovascular Diseases Center, Dongzhimen Hospital, Beijing University of Chinese Medicine, Beijing, China; ^5^Department of Cardiovascular, The First Affiliated Hospital of Guangzhou University of Chinese Medicine, Guangzhou, China; ^6^Cardiovascular Diseases Center, The Affiliated Hospital of Shanxi University of Chinese Medicine, Taiyuan, China

**Keywords:** Shenqisuxin granule, complex coronary artery disease, percutaneous coronary intervention, cardiorespiratory fitness, randomized controlled trial

## Abstract

**Introduction:**

The Shenqisuxin granule (SQSX), a novel Chinese herbal formula, has the effect of preventing in-stent restenosis and improving angiogenesis. We intend to evaluate the efficacy and safety of SQSX to provide a possible therapeutic strategy for complex coronary artery disease (CCAD) after percutaneous coronary intervention (PCI).

**Methods/design:**

The study is a multi-center, randomized, double-blinded, parallel, placebo-controlled trial. A total of 120 participants will be randomized 1:1 into the intervention group and the control group. Based on standardized treatment, the intervention group and control group will receive SQSX and placebo for 2 months, respectively. The primary outcomes, metabolic equivalents (METS) and peak oxygen uptake (Peak VO_2_), and the secondary outcomes, including other indicators of cardiorespiratory fitness (CRF), the European Quality of Life Questionnaire (EQ-5D-5L), the Seattle Angina Scale (SAQ), etc., will be assessed at baseline and 2 months ± 3 days. In addition, the survey scales will also be tested at 1 month ± 3 days. Trimethylamine N-oxide (TMAO), high-sensitivity C-reactive protein (hs-CRP), and gut microbiota features will be assessed at baseline and 2 months ± 3 days to probe possible mechanism. The major adverse cardiac and cerebrovascular events (MACCE) and bleeding events will be monitored until the 12-month follow-up.

**Discussion:**

This study is launched to assess the efficacy and safety of SQSX in CCAD after PCI and probe the possible mechanism.

**Clinical trial registration:**

China Clinical Trial Registry, ChiCTR2200060979, Registered on June 14, 2022.

## Introduction

Complex coronary artery disease (CCAD), a lesion type with great difficulty and a low success rate in revascularization, is accompanied by a higher rate of major adverse cardiac and cerebrovascular events (MACCE) (1, 2). The 10-year mortality rate of three-vessel disease, chronic total occlusion, and left main lesion after percutaneous coronary intervention (PCI) is up to 21, 27.6, and 28%, respectively, much higher than the 18.6% for general lesions (3, 4). Therefore, one of the most urgent issues now is certainly how to reduce the incidence of MACCE.

Regarded as the fifth vital sign, cardiorespiratory fitness (CRF) reflects the body's ability to supply and utilize oxygen with the support of the circulatory and respiratory systems (5). The decline in CRF is commonly observed across the course of coronary artery disease (CAD) (6, 7). Currently, CRF is widely used for cardiac rehabilitation advice and prognosis assessment in CAD (5, 8). As gold standard indicators of CRF, the metabolic equivalents (METS) and peak oxygen uptake (Peak VO_2_) are both closely associated with MACCE. A 1-MET increment is associated with 13 and 15% decrements in the risk of all-cause mortality and major adverse cardiac events, respectively (9). The readmission rates and all-cause mortality can be predicted by Peak VO_2_ (10). Therefore, CRF could be used as a sensitive and reliable surrogate indicator to assess the incidence of MACCE. Several survey scales, including the European Quality of Life Questionnaire (EQ-5D-5L), the Seattle Angina Scale (SAQ), the Fatigue Severity Scale (FSS), and the Hamilton Anxiety Scale (HAMA), are used for evaluating the quality of life from different perspectives (11–14). Furthermore, the disturbed intestinal flora is associated with several CAD risk factors and contributes to atherosclerosis (15). In different stages of CAD, it's commonly accompanied by significant characteristic changes in the intestinal flora (16–18). The level of trimethylamine N-oxide (TMAO) is positively associated with the SYNTAX score II to quantify the complexity of CAD and causes an increased risk of MACCE (19, 20). We speculate that the intestinal flora and their metabolites could be potential targets for pharmacological effect.

The Shenqisuxin granule (SQSX) is a novel Chinese herbal formula for CAD under patent protection (Chinese patent number ZL202010122712.6). Our previous in-stent restenosis study in minipig has confirmed that the ingredients of SQSX prevent in-stent restenosis by inhibiting vascular smooth muscle cell proliferation (21). In addition, it also improves angiogenesis by activating the phosphatidylinositol 3-hydroxykinase-protein kinase B (PI3K-Akt) pathway and increasing the mRNA levels of vascular endothelial growth factor (VEGF) (22). At a time when CCAD is still a conundrum in the field of cardiovascular disease, we intend to evaluate the efficacy and safety of SQSX to provide a possible therapeutic strategy.

## Methods/design

### Study design

Approved by the Ethics Committee of Xiyuan Hospital, China Academy of Chinese Medical Sciences (CACMS) (Version No. CCAL-YJFA-3.0, December 15, 2021), the multi-center, randomized, double-blinded, parallel, placebo-controlled trial has been registered with the Chinese Clinical Trials Registry (ChiCTR2200060979). It is conducted under the guidelines of Good Clinical Practice and the Helsinki Declaration (23, 24). It adheres to the Standard Protocol Items: Recommendations for Interventional Trials Statements (SPIRIT) (25). The Consolidated Standards of Reporting Trials Extension for Chinese Herbal Medicine Formulas 2017 (CONSORT-CHM Formulas 2017) recommendations are strictly followed (26).

**Figure 1:**
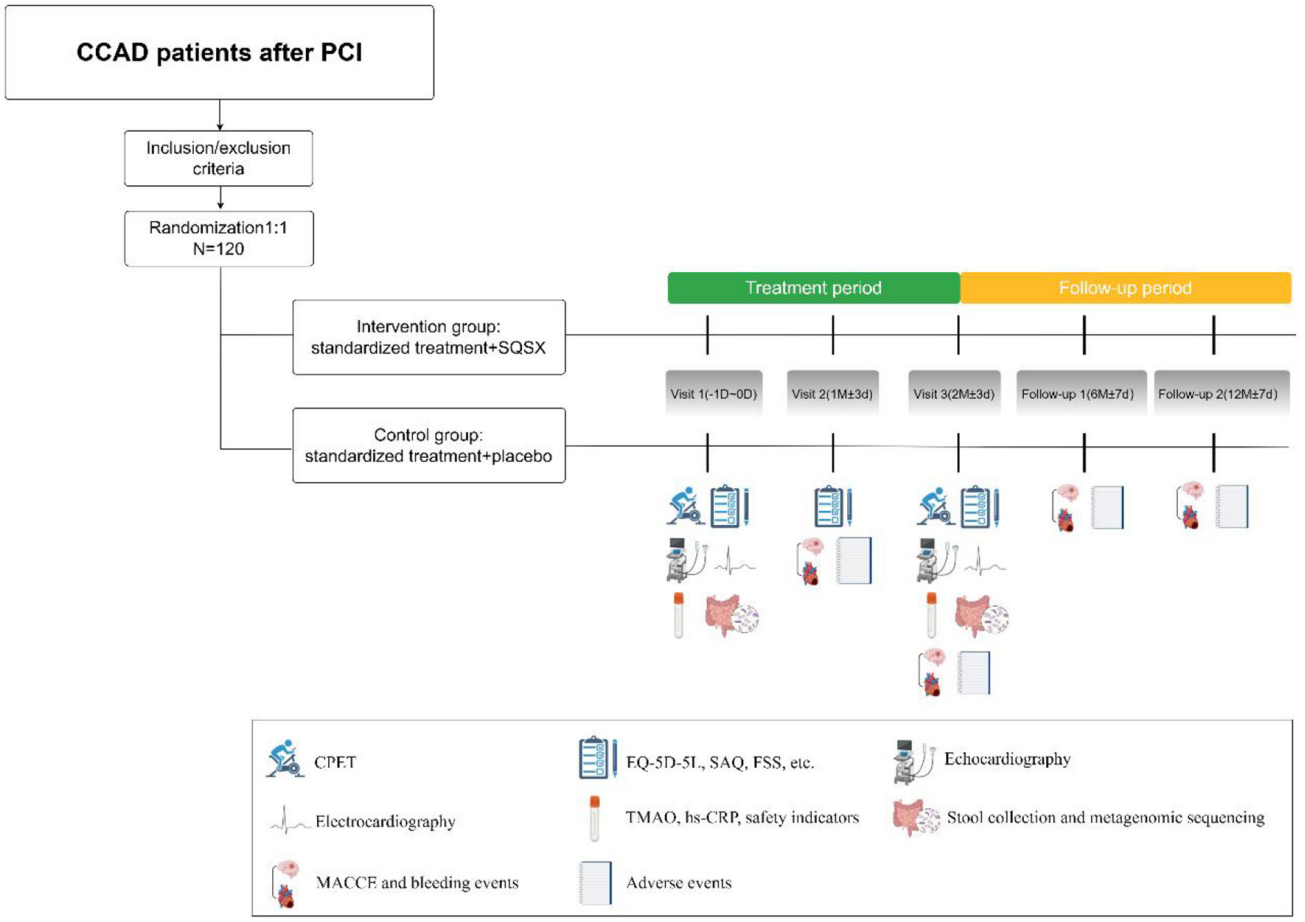
Flow diagram of the study.

The trial is being conducted in four centers in China, including Xiyuan Hospital of CACMS, Dongzhimen Hospital of Beijing University of Chinese Medicine, the First Affiliated Hospital of Guangzhou University of Chinese Medicine, and the Affiliated Hospital of Shanxi University of Chinese Medicine. It consists of two main periods: a 2-month treatment and a 10-month follow-up. A total of 120 participants will be randomized 1:1 into the intervention group and the control group and then receive their respective interventions for 2 months. Figure 1 depicts the study's design.

### Sample size estimation

The sample size was estimated based on METS. The study showed that after 8 weeks of cardiac rehabilitation, the METS increased from 3.9 to 4.6 in CAD patients after PCI (27). We assumed the mean METS value of 3.9 for the control group and 4.1 for the intervention group after treatment, with a standard deviation of 0.3 METS. With the type I error rate set at 0.05 and the type II error rate at 0.1, a sample size of 49 is required for each group with the use of PASS version 11. We will include 60 participants in each group, assuming that 20% of the participants are lost to follow up.

### Inclusion criteria

The study will include patients who satisfy all of the following inclusion criteria:

(1) Diagnosis of CCAD based on one or more of the following coronary artery lesions (28–30):

- Bifurcation lesions (side branch diameter >2.0 mm);
- Excessive tortuosity of proximal segment;
- Severe coronary artery calcification;
- Chronic total occlusion lesions (>3 months);
- Left main lesion;
- Aorto-ostial lesions;
- Diffuse lesions (>20 mm length);
- Multivessel coronary artery stenosis (≥2 two vessels);
- Extremely angulated lesions (>90° bend);
- In-stent restenosis.

(2) Diagnosis of stable angina according to the 2019 ESC guidelines (31);
(3) NYHA class I-III;
(4) At least one stent was implanted within 2 years;
(5) 18 years ≤ age ≤ 75 years;
(6) Diagnosed as the traditional Chinese medicine (TCM) syndrome of the Qi deficiency in the heart and spleen according to an array of specific symptoms, including chest tightness, abdominal distension, appetite loss, fatigue, and lassitude, with a pale tongue and a white greasy coating, etc., (32);
(7) No antibiotics, hormones, laxatives, antidiarrheals, or probiotics have been used within 3 months;
(8) Written informed consent is obtained.

### Exclusion criteria

Patients should be excluded if any of the following exclusion criteria are fulfilled:

(1) Renal insufficiency (serum creatinine >220 umol/l for males or >175 umol/l for females);
(2) Severe liver disease or elevated alanine transaminase (ALT) and aspartate transaminase (AST) (≥3 times upper limit of normal);
(3) Controlled systolic blood pressure or controlled diastolic blood pressure >160/100 mmHg, respectively;
(4) Glycosylated hemoglobin ≥9.5% or random blood glucose ≥13.7 mmol/L in diabetics;
(5) Women who are pregnant, are breastfeeding, or are planning to become pregnant;
(6) Acute or chronic severe cerebrovascular disease;
(7) Malignancies;
(8) Severe hematopoietic disorders;
(9) Severe mental illness;
(10) Intestinal inflammatory or malabsorptive disorders;
(11) Patients who have participated in other studies within 3 months.

### Withdrawal, dropout, and discontinuation

Participants can withdraw from the study whenever they want. The trial will be discontinued in the events of the following: (1) the continuation of the trial is detrimental to participants; (2) a serious adverse reaction occurs; (3) the participant's poor compliance. The cause of withdrawal will be recorded.

### Randomization and blinding

Stratified by center, sex, and age with a 1:1 allocation using random block sizes of 6, the randomization sequence was created using SAS software version 9.4. The investigators, patients and data analysts are blinded to the randomization sequence and treatment assignment. The emergency letter has been prepared, and the reason for opening the letter will be recorded if investigators break the blind.

### Intervention

All participants will receive standardized treatment based on the 2019 ESC guidelines (26), which includes antiischemic therapy, antiplatelet therapy, statins, etc. Furthermore, the intervention group and control group will receive SQSX and placebo for 2 months, respectively. The composition of SQSX is shown in Table 1. The placebo consists of 5% SQSX and 95% dextrin and is similar in odor and shape to the active granule. The SQSX and placebo were manufactured by the Department of Pharmaceutics of Xiyuan Hospital. The drug number is printed on the package of SQSX and placebo. The subjects will be instructed to take the granules after breakfast and supper (15 g per sachet, 1 sachet each time, twice daily). The granules will be supplied and/or recycled at each visit to verify compliance. During the trial, other medicines with an influence on the intestinal flora are forbidden.

**Table 1:** The composition of SQSX.

**Herb**	**Latin name**	**Dosage (g)**
Huang Qi	Astragali Radix	30
Dan Shen	Salviae Miltiorrhizae Radix Et Rhizoma	15
E zhu	Curcumae Rhizoma	9
Dang Gui	Angelicae Sinensis Radix	6
Bai Zhu	Atractylodis Macrocephalae Rhizoma	6
Huang Lian	Coptidis Rhizoma	5

### Study visits and follow-up

It begins with a 7-day lead-in period in which eligible participants sign informed consent. The baseline data will be gathered at Visit 1. Visits 2 and 3 will occur 1 month ± 3 days and 2 months ± 3 days following treatment, respectively. The follow-up will be conducted at 6 months ± 7 days and 12 months ± 7 days. Table 2 shows the evaluation schedule in detail.

**Table 2:** Measurement items and points of data capture.

	**Lead-in period**	**Treatment period**			**Follow-up period**	
	**Screening**	**Visit 1**	**Visit 2**	**Visit 3**	**Follow-up 1**	**Follow-up 2**
	**(-7D ~−1D)**	**(-1D ~0D)**	**(1M ±3d)**	**(2M ±3d)**	**(6M ±7d)**	**(12M ±7d)**
**Patients**						
Eligibility screen	×					
Informed consent	×					
General information^a^	×					
Medical history^b^	×					
Allocation	×					
**Intervention**						
Intervention group		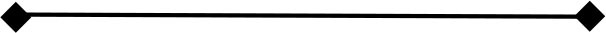				
Control group		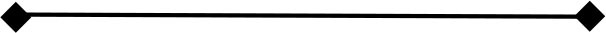				
**Outcomes**						
CPET^c^		×		×		
EQ-5D-5L		×	×	×		
SAQ		×	×	×		
FSS		×	×	×		
HAMA		×	×	×		
BSSS		×	×	×		
Echocardiography		×		×		
Electrocardiography		×		×		
TMAO		×		×		
hs-CRP		×		×		
Stool collection and metagenomic sequencing		×		×		
Safety indicators^d^		×		×		
MACCE and bleeding events^e^			×	×	×	×
**Others**						
Drug distribution/recycle		×	×	×		
Combined medication		×	×	×	×	×
AEs			×	×	×	×

a General information includes: name, date of birth, gender, ethnicity, employment status, etc.

b Medical history includes: current medical history of CCAD and past history.

c CPET: METS, Peak VO_2_, VO_2_/HR, AT, VE/VCO_2_ slope, etc. will be used to evaluate CRF.

d Safety indicators include: routine tests of blood, urine, stool, and the function of the liver, kidney, and coagulation.

e MACCE include: death, non-fatal myocardial infarction and ischemic stroke.

### Outcome measures

#### Primary outcome

The values of METS and Peak VO_2_ are measured to assess CRF after treatment. The condition of participants is evaluated before the test to avoid accidental events. The instruction is prepared for participants after informed consent is obtained. The cardiac and respiratory conditions of participants are monitored with breathing masks, electrodes, oximeters, etc. The test begins with a resting pulmonary function test, followed by a treadmill test. After pedaling without load for 1–3 min, the exercise load increases by 60W every minute until they are unable to continue testing (33). The professional physician is responsible for the interpretation of the result.

#### Secondary outcomes

The first of the secondary outcomes are other indicators of cardiopulmonary exercise testing (CPET), including the oxygen pulse (VO_2_/HR), the anaerobic threshold (AT), and the slope of ventilatory equivalent for carbon dioxide (VE/VCO_2_ slope), etc. The additional secondary outcomes are: (1) EQ-5D-5L; (2) SAQ; (3) FSS; (4) HAMA; (5) the blood stasis syndrome score (BSSS) (34); (6) echocardiography; (7) electrocardiography; (8) TMAO; (9) high-sensitivity C-reactive protein (hs-CRP); (10) MACCE and bleeding events. Furthermore, metagenomic sequencing will be performed to probe possible changes to the structure of intestinal flora after treatment. The plasma and stool samples will be collected and stored in the laboratory freezer (−80°C) for future TMAO testing and metagenomic sequencing.

#### Safety

Safety indicators, including routine tests of blood, urine, stool, and the function of the liver, kidney, and coagulation, will be performed at Visit 1 and Visit 3. All outcome measures are listed in Table 3 in detail.

**Table 3:** Primary, secondary and safety objectives.

**Objectives**	**Outcome measures**
**Primary objective**	
To determine whether SQSX is superior to placebo in improving patient's CRF.	Changes in METS and Peak VO_2_ after treatment.
**Secondary objectives**	
To determine whether SQSX is superior to placebo in improving patient's CRF.	Changes in other indicators of CPET, including VO_2_/HR, AT, and VE/VCO_2_ slope, etc. after treatment.
To compare the effect of SQSX vs. placebo on quality of life based on several survey scales.	Changes in scores measured by EQ-5D-5L, SAQ, FSS, and HAMA from baseline.
To compare the effect of SQSX vs. placebo on TCM syndrome.	Change in BSSS from baseline.
To assess cardiac structure and function with echocardiography and electrocardiography.	Results will be reported separately.
To explore the possible impact of SQSX on TMAO.	Change in TMAO after treatment.
To explore the possible impact of SQSX on hs-CRP.	Change in hs-CRP after treatment.
To depict intestinal flora features of CCAD and explore possible changes induced by SQSX.	Changes to the structure of intestinal flora after treatment using metagenomic sequencing.
To determine whether SQSX is superior to placebo in reducing the incidence of MACCE and bleeding events.	Time to the first occurrence of any of the components of this composite: (1) death; (2) non-fatal myocardial infarction; (3) ischemic stroke; (4) bleed.
**Safety objectives**	
To evaluate the safety of SQSX for CCAD after PCI.	(1) Changes in routine tests of blood, urine, stool, and the function of the liver, kidney, and coagulation; (2) AEs.

### Adverse events

Adverse events (AEs) refer to negative or unanticipated clinical medical events that occur during the trial. The CCAD may lead to acute coronary syndrome, malignant arrhythmia, and other AEs. Patients will be rescued promptly and actively if the above happens. The details of AEs will be recorded and reported to the ethics committee.

### Data collection and management

An independent data manager will be responsible for data input and management. The case report form will be carefully archived and then integrated into the data management system. The double input, data cleaning, manual checks, and other methods will be conducted to verify the data's validity, correctness, and completeness.

### Investigator training and quality control

A Standard Operating Procedure (SOP) is prepared for researchers in each center. All researchers have been trained to ensure standardization. The training includes screening of eligible participants, usage of survey scales, performance of CPET, etc. The WeChat group will be used to connect patients closely. To ensure the quality, regular supervision will be executed by the quality control group and the monitors appointed by Xiyuan Hospital.

### Statistical analysis

An independent statistician will be responsible for data analysis. The enrolling results and the causes of missing data will be detailed. The efficacy analysis will be performed based on the intent-to-treat (ITT) and per-protocol (PP) populations. The ITT set consists of all participants randomized, and the PP set includes participants who follow the protocol exactly with an adherence rate of at least 80%. The primary outcomes, METS and Peak VO_2_ after 2 months of treatment, and the secondary outcomes, the change values of VO_2_/HR, AT, VE/VCO_2_ slope, several survey scales (SAQ, EQ-5D-5L, HAMA, FSS, etc.), TMAO, and hs-CRP from baseline to each visit, are both quantitative data. Others, including the incidence of MACCE and bleeding events, are qualitative data. The quantitative data will be presented as means ± SD, 95% confidence intervals, median and interquartile range. For inter-group comparison, if both groups meet the normal distribution with equal variance, the Student *t*-test will be applied, otherwise, the Wilcoxon rank sum test will be used. For within-group comparison, the Wilcoxon signed rank test or paired *t*-test will be adopted. The qualitative data will be described using frequency and rate. The Fisher's exact probability test and the McNemar test will be applied for inter-group comparison and within-group comparison, respectively. Confounding factors will be balanced using analysis of covariance if necessary. The linear mixed-effects model will be used to analyze longitudinal data. For safety analysis, the details of AEs will be tabulated and the incidence will be compared further. The above analysis will be conducted based on SAS software version 9.4. In all analyses, a two-tailed *P*-value < 0.05 will be regarded as statistically significant.

## Discussion

The treatment for CCAD appears to be trapped. Currently, most studies on CCAD have focused on the strategies and medical devices for revascularization, including preferential choice of PCI or coronary artery bypass grafting (CABG), novel supreme drug-eluting stents, intravascular ultrasound-guided treatment, etc., (25, 35–37). Even with the help of state-of-the-art revascularization, the improvement in myocardial ischemia is usually regional and limited. With strict adherence to evidence-based secondary prevention of CAD after PCI, the severe threats of high incidence of MACCE and low quality of life are still facing patients with CCAD (38, 39). To our knowledge, there are no viable therapies or preventative strategies for these conditions. Our previous studies have confirmed that SQSX has the favorable effects of improving angiogenesis and preventing in-stent restenosis. The Chinese herbal formula with multi-target characteristics has the potential to provide a promising therapeutic strategy for CCAD after PCI. Thus, tailored to the unique population of CCAD, this multi-center, randomized, double-blinded, parallel, placebo-controlled clinical study uses CPET, several survey scales, and other methods to evaluate the efficacy and safety of SQSX.

There are two limitations to this work. First, as a surrogate indicator, CRF doesn't directly represent the real influence on MACCE. In the future, the clinical endpoint of MACCE will be used as the primary outcome measure to conduct clinical research. Second, CCAD includes a variety of lesions, such as multivessel disease, left main lesions, chronic total occlusion lesions, etc. The differences between various lesions of CCAD may affect the result of the trial. Thus, the subgroup analysis will be performed to minimize the impact of these differences.

To conclude, this study is launched to evaluate the efficacy and safety of SQSX in CCAD after PCI and probe the possible mechanism.

## Author contributions

PW designed the study under the instruction of KC. XW and MY drafted the manuscript and participated in the preparation of the study. XP, HW, ZH, RX, JP, YL, and XW are responsible for the execution of the trial. SS, YD, and JL reviewed and polished the manuscript. All authors read and approved the final manuscript.

## Funding

This trial is supported financially by the CACMS Innovation Fund (No. CI2021A00905).

## Conflict of interest

The authors declare that the research was conducted in the absence of any commercial or financial relationships that could be construed as a potential conflict of interest.

## Publisher's note

All claims expressed in this article are solely those of the authors and do not necessarily represent those of their affiliated organizations, or those of the publisher, the editors and the reviewers. Any product that may be evaluated in this article, or claim that may be made by its manufacturer, is not guaranteed or endorsed by the publisher.

## Abbreviations

AEs: adverse events
ALT: alanine transaminase
AST: aspartate transaminase
AT: anaerobic threshold
BSSS: Blood Stasis Syndrome Score
CABG: coronary artery bypass grafting
CCAD: complex coronary artery disease
CACMS: China Academy of Chinese Medical Sciences
CAD: coronary artery disease
CONSORT-CHM Formulas 2017: Consolidated Standards of Reporting Trials Extension for Chinese Herbal Medicine Formulas 2017
CPET: cardiopulmonary exercise testing
CRF: cardiorespiratory fitness
EQ-5D-5L: European Quality of Life Questionnaire
ESC: European Society of Cardiology
FSS: Fatigue Severity Scale
HAMA: Hamilton Anxiety Scale
hs-CRP: high-sensitivity C-reactive protein
MACCE: major adverse cardiac and cerebrovascular events
METS: metabolic equivalents
NYHA: New York Heart Association
PCI: percutaneous coronary intervention
Peak VO_2_: peak oxygen uptake
PI3K-Akt: phosphatidylinositol 3-hydroxykinase-protein kinase B
SAQ: Seattle Angina Scale
SOP: Standard Operating Procedure
SPIRIT: Standard Protocol Items: Recommendations for Interventional Trials Statements
SQSX: Shenqisuxin granule
TCM: traditional Chinese medicine
TMAO: trimethylamine N-oxide
VEGF: vascular endothelial growth factor
VE/VCO_2_ slope: slope of ventilatory equivalent for carbon dioxide
VO_2_/HR: oxygen pulse.
